# Rapid growth of a pit-free (011) β-Ga_2_O_3_ homoepitaxial layer with very low residual Si and Cl concentrations by HCl-based halide vapor phase epitaxy

**DOI:** 10.1080/14686996.2026.2751773

**Published:** 2026-10-22

**Authors:** Yuichi Oshima, Takayoshi Oshima

**Affiliations:** Research Center for Electronic and Optical Materials, National Institute for Materials Science, Tsukuba, Japan

**Keywords:** β-Ga_2_O_3_, HVPE, impurity

## Abstract

We report the rapid growth of pit-free (011) β-Ga_2_O_3_ homoepitaxial layers with very low residual [Si] and [Cl] by HCl-based halide vapor phase epitaxy (HCl-HVPE) in which GaCl was generated from Ga and HCl. Along with the GaCl and O_2_ growth precursors, additional HCl was introduced directly into the growth zone to suppress parasitic gas-phase reactions. The effects of O_2_ and additional HCl partial pressures on residual impurity concentrations were systematically investigated. The [Si] decreased sharply with increasing O_2_ partial pressure before reaching saturation, whereas it increased monotonically with additional HCl partial pressure and fell below the secondary ion mass spectrometry detection limit (1 × 10^15^ cm^−3^) in the absence of additional HCl. Thermodynamic analysis of SiO_2_ etching by H_2_ and HCl qualitatively reproduced these trends and suggested that residual Si originates predominantly from HCl-induced etching of quartz components in the source zone in the reactor. Even without additional HCl, increasing the GaCl supply enabled a growth rate of 13 μm/h, comparable to that previously achieved with additional HCl, while maintaining [Si] below the detection limit and [Cl] below 2 × 10^15^ cm^−3^, without particle-induced surface degradation. Furthermore, no pit formation was observed in the absence of additional HCl, and the pits were found to originate from substrate pre-etching by additional HCl before growth. These findings demonstrate that HCl-HVPE enables rapid homoepitaxy of pit-free (011) β-Ga_2_O_3_ layers with very low residual impurity concentrations, offering a promising route toward cost-effective vertical β-Ga_2_O_3_ power devices.

## Impact Statement

This study identified the origins of residual Si contamination and pit formation in HCl-HVPE and demonstrates rapid growth of pit-free (011) β-Ga_2_O_3_ homoepitaxial layers with very low residual donor concentrations.

## GRAPHICAL ABSTRACT

**Figure:**
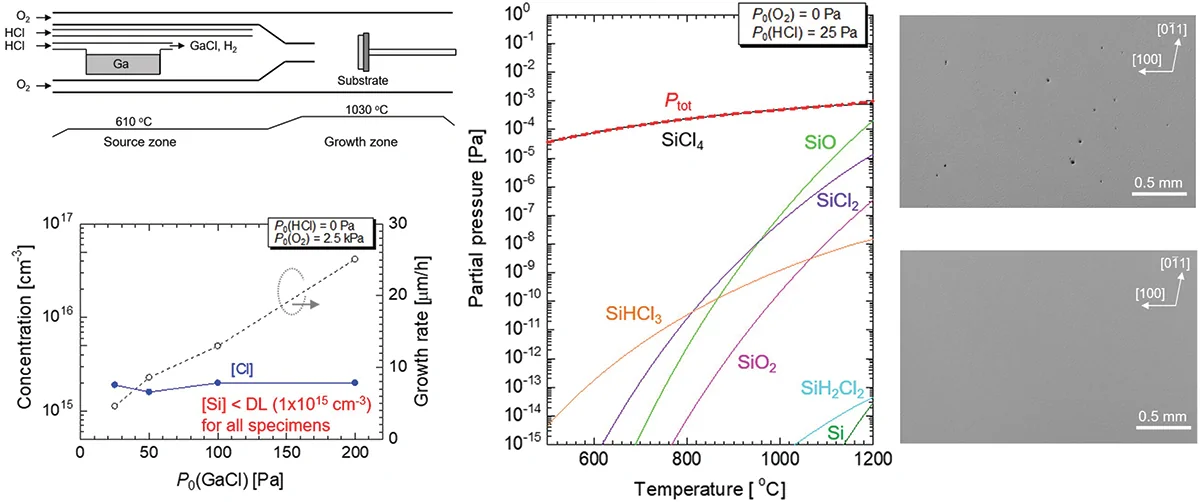

## Introduction

β-Ga_2_O_3_ is the most thermodynamically stable polymorph of Ga_2_O_3_ and possesses an ultra-wide bandgap of 4.5–4.9 eV [1,2]. Owing to its wide bandgap, β-Ga_2_O_3_ is expected to exhibit a high critical electric field [3]. Furthermore, large-area, high-quality single-crystal substrates grown by melt-growth techniques are commercially available, enabling homoepitaxial growth [4–10]. These advantages have established β-Ga_2_O_3_ as one of the most promising semiconductor materials for next-generation high-performance power electronics. Indeed, high-performance prototype devices, including Schottky barrier diodes (SBDs) [11–14] and metal–oxide–semiconductor field-effect transistors (MOSFETs) [15–19], have already been demonstrated.

For high-voltage β-Ga_2_O_3_ power devices, the drift layer is required to be both thick and lightly doped. For example, realizing a device with a breakdown voltage of approximately 10 kV requires a drift layer with a thickness of 30–50 μm and a carrier concentration as low as 5 × 10^15^ − 1 × 10^16^ cm^−3^ [20]. Therefore, an epitaxial growth technique for high-voltage β-Ga_2_O_3_ power devices must provide both a high growth rate for cost-effective fabrication and a residual carrier concentration sufficiently lower than the required carrier concentration. Halide vapor phase epitaxy (HVPE) is a promising epitaxial growth technique potentially capable of meeting these requirements.

HVPE of β-Ga_2_O_3_ can be broadly classified into two types according to the method used to generate the GaCl precursor. One approach employs the reaction of metallic Ga with Cl_2_ gas to produce GaCl and is hereafter referred to as Cl_2_-HVPE. A notable advantage of Cl_2_-HVPE is its extremely low residual [Si], which is typically below the secondary ion mass spectrometry (SIMS) detection limit (DL), for example, <5 × 10^15^ cm^−3^ [21]. Consequently, Cl_2_-HVPE has been employed in most demonstrations of vertical β-Ga_2_O_3_ power devices [12–14,16,17,19]. However, increasing the precursor concentration to achieve higher growth rates tends to promote parasitic gas-phase reactions, resulting in particle formation [22]. As a result, commercially available (001) β-Ga_2_O_3_ epitaxial wafers are typically grown at a relatively low growth rate of approximately 4 μm/h [20].

The other type of HVPE employs the reaction of metallic Ga with HCl gas to generate the GaCl precursor and is hereafter referred to as HCl-HVPE to distinguish it from Cl_2_-HVPE according to the chlorine source. A characteristic feature of HCl-HVPE is the simultaneous generation of H_2_ as a by-product during GaCl formation. In GaN grown by HCl-HVPE, relatively high residual [Si] (typically on the order of 10^17^ cm^−3^ [23]) have been widely reported. This has been attributed to etching of the quartz reactor components, which are extensively used in HVPE reactors, by H-containing gases (H_2_, HCl, and NH_3_), resulting in the generation of Si-containing gaseous species. Indeed, it has been reported that the residual [Si] can be reduced to below 5 × 10^14^ cm^−3^ by eliminating quartz components from the HVPE reactor [23]. By analogy with these findings for GaN, HCl-HVPE has generally been considered more susceptible than Cl_2_-HVPE to Si contamination in β-Ga_2_O_3_ epilayers [24]. On the other hand, HCl-HVPE generally enables higher growth rates than Cl_2_-HVPE [25–27]. This is presumably because the H_2_ generated during growth, which also acts as an etchant for β-Ga_2_O_3_, suppresses particle formation caused by parasitic reactions between the source gases. Furthermore, the growth rate in HCl-HVPE can be further increased by introducing additional HCl into the growth zone [26,28,29]. For example, we routinely achieve a high growth rate of 24 μm/h for (001) β-Ga_2_O_3_ growth using HCl-HVPE with additional HCl [26,28].

To date, (001)-oriented substrates have been predominantly used for the epitaxial growth of β-Ga_2_O_3_ drift layers for vertical power devices. This is mainly because the (001) plane is parallel to the [010] pulling direction used in the edge-defined film-fed growth (EFG) method, facilitating the fabrication of large-area substrates, and because it generally allows higher HVPE growth rates than other crystal orientations [30]. However, a common drawback of (001) homoepitaxy by both types of HVPE is the relatively high residual [Cl], with typical values around 1 × 10^16^ cm^−3^ [20,26,31]. One promising approach to overcoming this issue is to employ the (011) orientation. For both Cl_2_-HVPE and HCl-HVPE, the residual [Cl] in (011) epilayers has been reported to be approximately one order of magnitude lower than that in (001) epilayers [26,32]. However, the growth rate on the (011) plane is only about 60% of that on the (001) plane and is as low as approximately 2 μm/h for Cl_2_-HVPE [32]. Nevertheless, several promising vertical β-Ga_2_O_3_ power devices based on (011) epilayers have been demonstrated [19,33,34]. For example, Wakimoto et al. reported a FinFET with a breakdown voltage exceeding 10 kV incorporating an 85-μm-thick drift layer grown by Cl_2_-HVPE [19].

In contrast, HCl-HVPE with additional HCl has been reported to achieve a much higher growth rate of 14 μm/h than that reported for Cl_2_-HVPE, while maintaining a low residual [Cl] of 1.7 × 10^15^ cm^−3^. However, the residual [Si] remains relatively high at 2 × 10^16^ cm^−3^ [26]. Another issue is the formation of pits on the epilayer surface at a density of approximately 10^3^–10^4^ cm^−2^, a phenomenon that has not been reported for Cl_2_-HVPE [26]. These pits extend nearly to the epilayer/substrate interface, suggesting that their origin is associated with the epilayer/substrate interface.

If the residual [Si] and pit formation on the (011) surface can be effectively suppressed, HCl-HVPE could become a promising epitaxial growth technique for β-Ga_2_O_3_ power devices by combining high growth rates with the intrinsic advantage of low Cl incorporation. In this work, we investigate the origins of residual Si incorporation and pit formation in HCl-HVPE. Furthermore, we demonstrate rapid homoepitaxial growth of pit-free (011) β-Ga_2_O_3_ with a residual [Si] below the SIMS detection limit while maintaining the low residual [Cl] characteristic of the (011) orientation.

## Experimental methods

(011) β-Ga_2_O_3_ homoepitaxial growth was carried out by atmospheric-pressure HVPE at 1030°C without intentional doping on conductive Sn-doped (011) β-Ga_2_O_3_ single-crystal substrates grown by the EFG method (Novel Crystal Technology, Inc.) using a laboratory-built quartz reactor. A schematic illustration of the HVPE reactor is shown in Figure 1. Not only the main reactor tube but also all internal components were made of quartz. High-purity O_2_ gas (>99.99995%) was used as the oxygen source. GaCl precursor gas was generated in the source zone maintained at 610°C by reacting metallic Ga (>99.99999%) with source HCl gas (>99.999%) in the Ga boat. Under these conditions, the source HCl was essentially completely consumed and converted into GaCl and H_2_. In addition to the precursor gases, the reactor was equipped with a supply line for additional HCl gas to suppress parasitic reactions between the source gases [26,29]. Unlike the source HCl, the additional HCl bypassed the Ga boat without reacting with metallic Ga and was introduced directly into the growth zone. N_2_ gas (dew point < −110 °C) was used as the carrier gas for all gas streams. The GaCl and additional HCl gas streams were transported independently of the O_2_ gas stream and mixed only in the growth zone, where β-Ga_2_O_3_ growth took place. No intentional post-growth annealing was performed.

**Figure 1.**
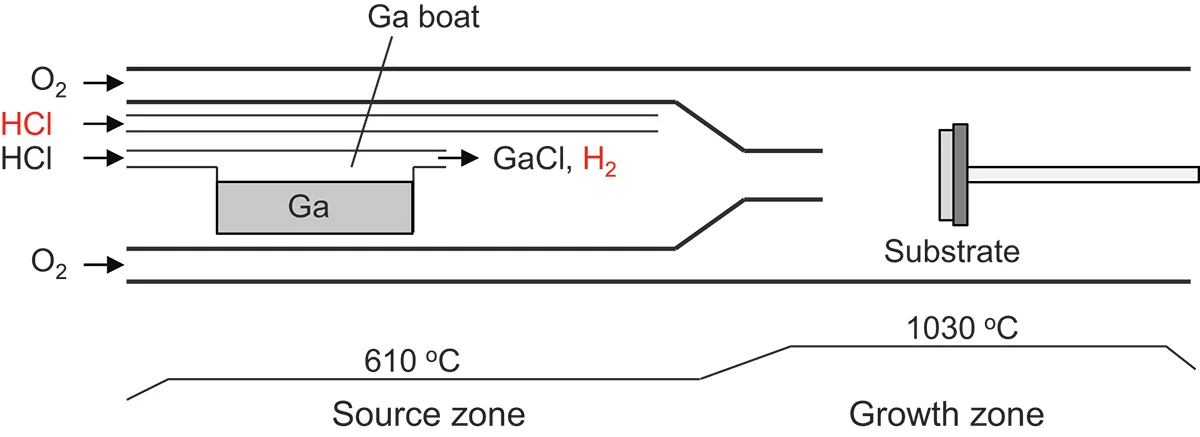
Schematic of the HVPE reactor used in this study. Carrier gas flows are omitted for clarity.

During the temperature ramp-up, O_2_ was supplied at the same partial pressure as that used during the growth step. After the reactor temperature had stabilized, the GaCl supply was initiated to start epitaxial growth. When additional HCl was used, its supply was started 5 min before the introduction of GaCl. Consequently, the β-Ga_2_O_3_ substrates were subjected to a slight etching treatment prior to growth.

The growth rate was determined from the substrate weight change before and after epitaxial growth. The surface morphology was characterized by Nomarski optical microscopy using BX51 (OLYMPUS, Japan) and scanning electron microscopy (SEM) using SU8230 (Hitachi, Japan). Impurity concentrations were measured by SIMS.

To confirm that the residual carrier concentration was sufficiently reduced, a SBD consisting of an unintentionally doped homoepitaxial layer grown on a conductive Sn-doped (011) β-Ga_2_O_3_ substrate was fabricated. Its current–voltage (*J–V*) and capacitance–voltage (*C–V*) characteristics were measured using a semiconductor parameter analyzer B1500A (Keysight, US). The *C*–*V* measurement was performed at an AC frequency of 1 MHz, assuming a relative dielectric constant of 10 for β-Ga_2_O_3_. For the cathode, Ti (30 nm)/Pt (20 nm)/Au (70 nm) contacts were deposited by electron-beam (EB) evaporation, followed by rapid thermal annealing (RTA) at 550°C for 1 min in N_2_ gas flow. The ohmic behavior of the backside contact was confirmed by the linear *I*–*V* characteristics measured between adjacent cathode electrodes. The Schottky anode consisted of a 50-nm-thick Au electrode with an area of 2.3 × 10^−3^ cm^2^ was deposited by EB evaporation.

## Results and discussion

Figure 2(a) shows the dependences of the residual [Si] and [Cl], and growth rate on the supplied O_2_ partial pressure, *P*_0_(O_2_). Here, *P*_0_(*X*) denotes the supplied partial pressure of gas species *X*. Note that *P*_0_(HCl) hereafter refers to the partial pressure of the additional HCl rather than that of the source HCl. Throughout this experiment, both *P*_0_(GaCl) and *P*_0_(HCl) were fixed at 25 Pa. The growth rate increased with increasing *P*_0_(O_2_), gradually approaching saturation. The residual [Si] was 1.5 × 10^16^ cm^−3^ at the lowest *P*_0_(O_2_) investigated but decreased rapidly to 3.2 × 10^15^ cm^−3^, approximately one-fifth of the initial value, at *P*_0_(O_2_) = 0.625 kPa. A further increase in *P*_0_(O_2_) produced little change in the residual [Si]. In contrast, the residual [Cl] exhibited a slight increase with increasing *P*_0_(O_2_), while remaining as low as 1.5 × 10^15^ cm^−3^ even at the highest *P*_0_(O_2_) investigated. Considering that Cl is generally believed to occupy O sites in β-Ga_2_O_3_ [35], one might expect the Cl incorporation to decrease with increasing *P*_0_(O_2_). However, the opposite trend was observed. This result suggests that Cl incorporation is influenced by mechanisms other than simple competition between O and Cl for O lattice sites. The origin of this behavior remains unclear at present and requires further investigation.

**Figure 2.**
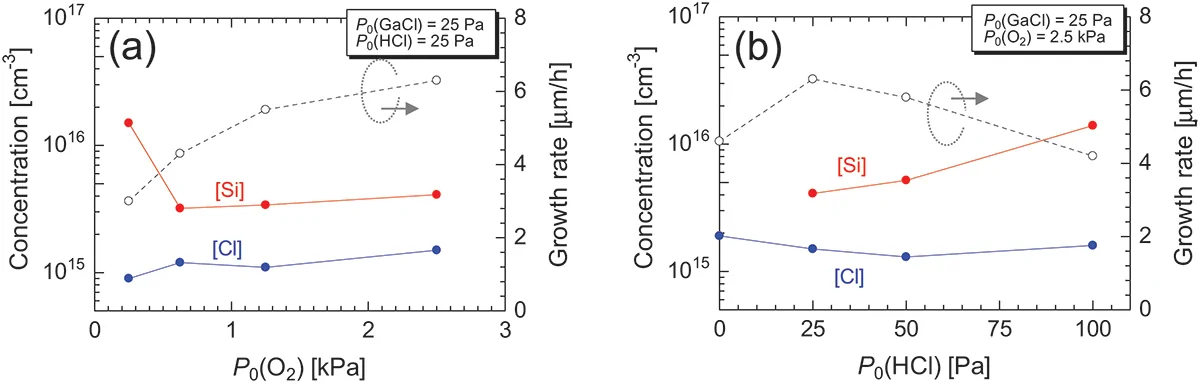
Dependences of the residual [Si] and [Cl], and growth rate on (a) *P*_0_(O_2_) and (b) *P*_0_(HCl).

Figure 2(b) shows the dependences of the residual [Si] and [Cl], and the growth rate on *P*_0_(HCl). In this experiment, *P*_0_(GaCl) and *P*_0_(O_2_) were fixed at 25 Pa and 2.5 kPa, respectively. The growth rate initially increased with increasing *P*_0_(HCl) and then gradually decreased. This behavior is considered to result from two competing effects. At low *P*_0_(HCl), the additional HCl suppresses parasitic gas-phase reactions, thereby improving the transport efficiency of the source species to the substrate. At higher *P*_0_(HCl), however, the etching of β-Ga_2_O_3_ on the substrate becomes dominant, resulting in a reduction in the net growth rate. When no additional HCl was supplied, the residual [Si] was below the SIMS detection limit (1 × 10^15^ cm^−3^); therefore, this data point is not included in Figure 2(b). The residual [Si] increased monotonically with increasing *P*_0_(HCl) and reached 1.4 × 10^16^ cm^−3^ at *P*_0_(HCl) = 100 Pa. In contrast, the residual [Cl] exhibited no clear dependence on *P*_0_(HCl) and remained nearly constant in the range of (1.3–1.9) × 10^15^ cm^−3^.

As shown in Figure 2(a), the residual [Si] can be substantially reduced by increasing *P*_0_(O_2_). This reduction is most likely attributable to the suppression of SiO_2_ reduction by the H-containing species, H_2_ and HCl, in the presence of O_2_. A notable feature is that the residual [Si] did not decrease continuously with increasing *P*_0_(O_2_). Instead, it dropped sharply with a relatively small increase in *P*_0_(O_2_) and then remained nearly constant. This behavior suggests that even a relatively low O_2_ partial pressure is sufficient to effectively suppress the reduction of SiO_2_.

The monotonic increase in the residual [Si] with increasing *P*_0_(HCl), shown in Figure 2(b), further suggests that HCl plays a significant role in etching SiO_2_ and thereby enhances Si contamination. It should be noted that, even in the absence of additional HCl, H_2_ generated during GaCl formation was still present in the reactor at a partial pressure of one-half that of *P*_0_(GaCl) (12.5 Pa). If H_2_ had an etching effect on SiO_2_ comparable to or greater than that of HCl under the present growth conditions, a detectable concentration of Si would also be expected even without additional HCl. However, the residual [Si] remained below the SIMS detection limit. These results therefore suggest that, under the present growth conditions, the effective etching capability of H_2_ toward SiO_2_ was weaker than that of HCl.

To elucidate the experimentally observed behavior of the residual [Si], the thermodynamic driving forces for SiO_2_ etching by H_2_ and HCl were evaluated, and their dependences on temperature and *P*_0_(O_2_) were analyzed. The thermodynamic model and calculation procedure are described below. Assuming that the SiO_2_ etching rate is governed primarily by the equilibrium generation of volatile Si-containing species, the thermodynamic driving force for SiO_2_ etching is expected to correlate with the total equilibrium partial pressure of these species at the SiO_2_ surface. Accordingly, the following quantity was adopted as an indicator of the etching driving force:

(1) $$P_{\mathrm{tot}}=\underset{i}{\sum }n_{i}P\left(Si_{n_{i}}X_{i}\right)$$

where $P\left(Si_{n_{i}}X_{i}\right)$ is the equilibrium partial pressure of a gaseous species $Si_{n_{i}}X_{i}$.

The equilibrium partial pressures of all gaseous species were calculated using the thermodynamic equilibrium software CatCalc (Research Institute of Computational Thermodynamics, Inc.), which determines the equilibrium gas composition by minimizing the Gibbs free energy of the system [36].

For the analysis of SiO_2_ etching by H_2_, the following gaseous species were included in the equilibrium calculation: Si, Si_2_, Si_3_, SiH_4_, SiO, SiO_2_, Si(OH)_4_, SiH_2_O_3_, H_2_, H_2_O, H_2_O_2_, O_2_, and O_3_, together with inert carrier gas. For the analysis of SiO_2_ etching by HCl, the following gaseous species were considered: Si, Si_2_, Si_3_, SiH_4_, SiO, SiO_2_, Si(OH)_4_, SiH_2_O_3_, SiCl_2_, SiCl_4_, SiH_2_Cl_2_, SiH_3_Cl, SiHCl_3_, H_2_, H_2_O, H_2_O_2_, O_2,_ O_3_, Cl_2_, Cl_4_, Cl_2_O, ClO_2_, HCl, HClO, and HClO_4_, together with inert carrier gas.

For the H_2_ etching calculations, *P*_0_(H_2_) was fixed at 12.5 Pa, corresponding to the H_2_ generated as a by-product during GaCl formation from metallic Ga and source HCl under the growth conditions used for the experiments shown in Figure 2(a,b), where *P*_0_(GaCl) was 25 Pa. For the HCl etching calculations, *P*_0_(HCl) was fixed at 25 Pa, corresponding to the experimental conditions used in Figure 2(a). *P*_0_(O_2_), was varied from 0 to 2.5 kPa to cover the experimental growth conditions.

Figure 3(a,b) show the calculated temperature dependences of the equilibrium partial pressures of individual volatile Si-containing species, $Si_{n_{i}}X_{i}$, and the etching driving force indicator, *P*_tot_, for SiO_2_ etching by H_2_ (*P*_0_(H_2_) = 12.5 Pa) and HCl (*P*_0_(HCl) = 25 Pa), respectively, under *P*_0_(O_2_) = 0. The vertical dashed lines indicate the source-zone temperature (610 °C) and the growth-zone temperature (1030 °C). The equilibrium partial pressures of the Si-containing species not shown are below the plotting range.　The dominant volatile Si-containing species generated by H_2_ and HCl etching are SiO and SiCl_4_, respectively, and their equilibrium partial pressures almost completely overlap with the corresponding *P*_tot_ curves. A notable feature is that the temperature dependence of the etching driving force is much weaker for HCl than for H_2_. Consequently, although the HCl-induced etching driving force is comparable to that for H_2_ at the growth-zone temperature of 1030°C, it is much greater at the source-zone temperature of 610°C.

**Figure 3.**
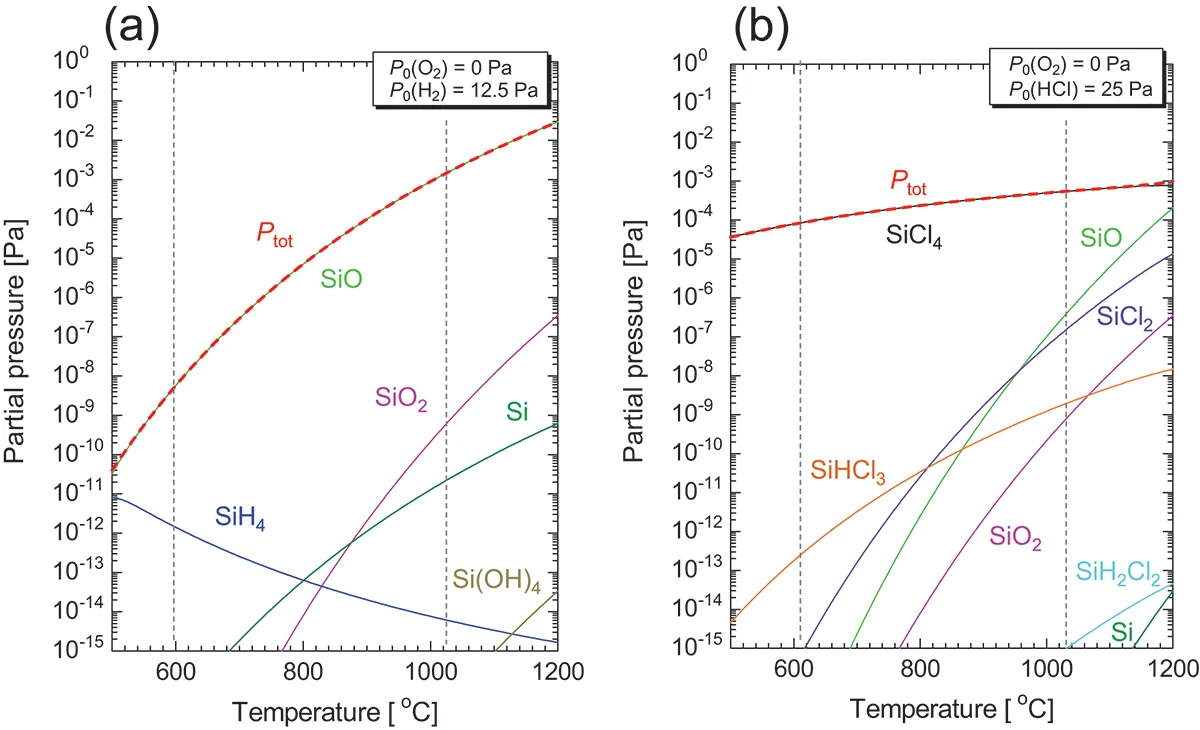
Calculated temperature dependences of the equilibrium partial pressures of Si-containing gaseous species and the etching driving force indicator, *P*_tot_, for SiO_2_ etching by (a) H_2_ and (b) HCl.

Figure 4(a,b) show the calculated temperature dependences of the etching driving force indicator, *P*_tot_, for SiO_2_ etching by H_2_ (*P*_0_(H_2_) = 12.5 Pa) and HCl (*P*_0_(HCl) = 25 Pa), respectively, in the presence of O_2_. For both etchants, *P*_tot_ decreases markedly with O_2_ supply. In Figure 4(a), the *P*_tot_ curves for *P*_0_(O_2_) ≥12.5 Pa almost completely overlap. It should also be noted that the dominant volatile Si-containing species depend on *P*_0_(O_2_). Under the standard experimental condition of *P*_0_ (O_2_) = 2.5 kPa, Si(OH)_4_ becomes the dominant Si-containing species for both H_2_ and HCl etching, in contrast to the O_2_-free case. The calculated temperature dependences of the equilibrium partial pressures of individual Si-containing species and *P*_tot_ for various *P*_0_(O_2_) values are presented in Figs. S1 and S2.

**Figure 4.**
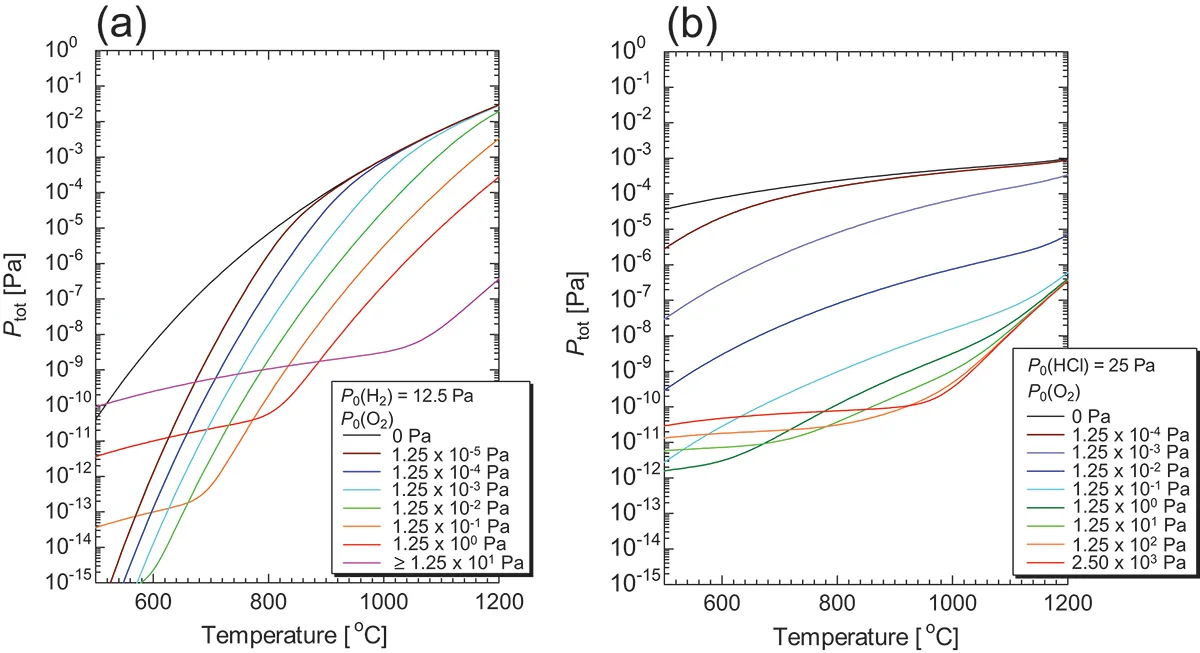
Calculated temperature dependences of the etching driving force indicator, *P*_tot_, at various *P*_0_(O_2_): (a) H_2_ etching and (b) HCl etching.

Figure 5(a,b) show the calculated dependences of *P*_tot_ on *P*_0_(O_2_) at 1030°C for SiO_2_ etching by H_2_ (*P*_0_(H_2_) = 12.5 Pa) and HCl (*P*_0_(HCl) = 25 Pa), respectively. In both cases, *P*_tot_ decreases sharply even with a small O_2_ supply. For example, in the H_2_ etching calculation, supplying O_2_ at only one-tenth of the H_2_ partial pressure (*P*_0_(O_2_) = 1.25 Pa) reduces *P*_tot_ to less than one-thousandth of its value without O_2_. These calculated results provide a qualitative explanation for the experimentally observed sharp decrease in the residual [Si] with a relatively small increase in *P*_0_(O_2_), followed by the appearance of a plateau, as shown in Figure 2(a).

**Figure 5.**
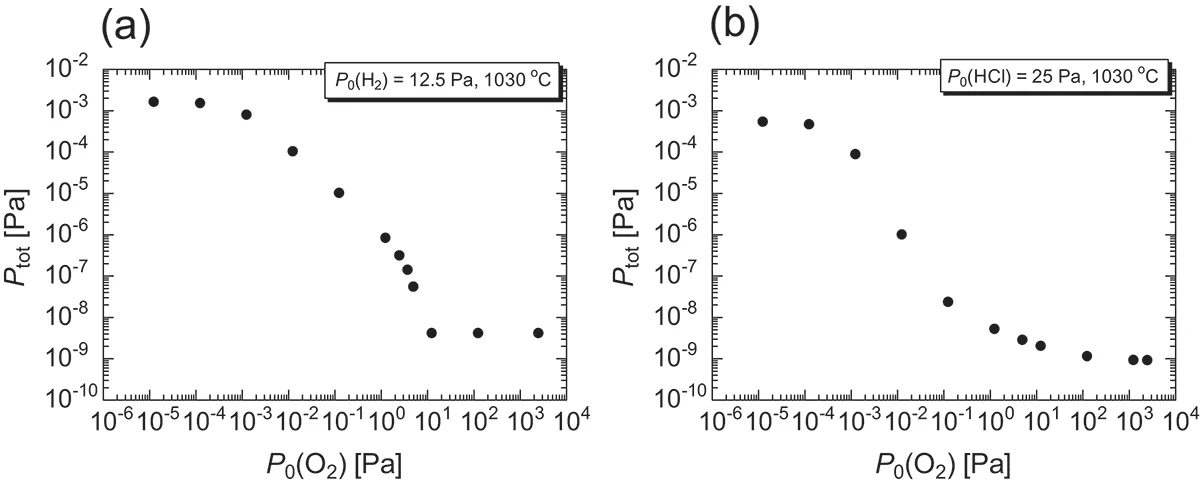
Calculated dependences of the etching driving force indicator, *P*_tot_, on *P*_0_(O_2_) at 1030°C for (a) H_2_ etching and (b) HCl etching.

Table 1 compares the calculated etching driving forces for SiO_2_ by H_2_ and additional HCl in the source zone and the growth zone of the HVPE reactor. In the source zone, the etching driving force for additional HCl is much greater than that for H_2_. This is because the etching driving force of HCl exhibits a much weaker temperature dependence than that of H_2_, allowing HCl to maintain a high etching capability even at the relatively low source-zone temperature of 610°C.

**Table 1.** Comparison of the etching driving force indicator, *P*_tot_, for H_2_ and HCl in the source and growth zones of the HVPE reactor.

Etchant	Source zone (610 °C) *P*_0_(O_2_) = 0 kPa	Growth zone (1030 °C) *P*_0_(O_2_) = 25 kPa
H_2_ *P*_0_(H_2_) = 12.5 Pa	8.8 × 10^−14^ Pa	4.1 × 10^−14^ Pa
HCl *P*_0_(HCl) = 25 Pa	8.4 × 10^−10^ Pa	9.1 × 10^−15^ Pa

In contrast, the etching driving forces in the growth zone are much smaller than that for additional HCl in the source zone for both etchants because of the strong suppression by O_2_. These results therefore suggest that SiO_2_ etching in the HVPE reactor is dominated by additional HCl in the source zone. This mechanism provides a qualitative explanation for the strong dependence of the residual [Si] on *P*_0_(HCl) observed in Figure 2(b).

The experimental results described above demonstrated that the residual [Si] in (011) β-Ga_2_O_3_ homoepitaxial layers can be reduced below the SIMS detection limit by eliminating the supply of additional HCl. However, under these conditions, the growth rate was limited to 4.6 μm/h for *P*_0_(GaCl) = 50 Pa, which is considerably lower than the reported value of 14 μm/h for HVPE with additional HCl [26]. Therefore, the growth rate was increased by increasing *P*_0_(GaCl) while maintaining *P*_0_(O_2_) at 2.5 kPa. Figure 6(a) shows the dependences of the growth rate, the residual [Si] and [Cl] on *P*_0_(GaCl). The labels (b) and (c) correspond to the SEM images shown in Figure 6(b,c), respectively. At *P*_0_(GaCl) = 100 Pa [label (b)], the growth rate reached 13 μm/h, comparable to that reported for HVPE with additional HCl, while no degradation of the surface morphology was observed, as shown in Figure 6(b). When *P*_0_(GaCl) was further increased to 200 Pa [label (c)], the growth rate increased to 25 μm/h. However, numerous particles were observed on the epilayer surface, as shown in Figure 6. These particles were most likely generated by parasitic reactions between GaCl and O_2_ under the high precursor concentration. To further increase the growth rate while suppressing particle formation, two approaches appear promising: supplying a small amount of additional HCl while maintaining the residual [Si] below the acceptable level, or employing reduced-pressure growth.

**Figure 6.**
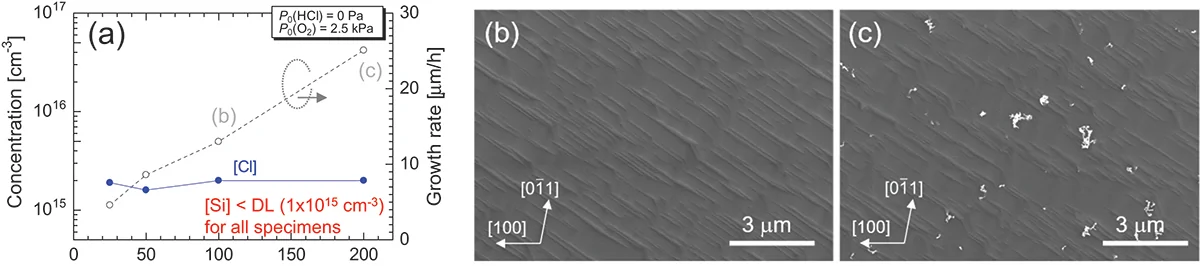
(a) Dependences of the residual [Cl] and growth rate on *P*_0_(GaCl). The residual [Si] was below the SIMS detection limit under all growth conditions. (b), (c) corresponding plan-view SEM images of the epilayer surfaces grown at *P*_0_(GaCl) = 100 and 200 Pa, respectively.

As also shown in Figure 6(a), no significant increase in the residual [Cl] was observed even at the highest growth rates, and the residual [Si] remained below the SIMS detection limit (1 × 10^15^ cm^−3^) for all samples. In contrast, for the (001) orientation, a significant increase in the residual [Cl] has been reported for *P*_0_(GaCl) increase [26]. This difference suggests that, in addition to thermodynamic factors, crystal-orientation-dependent kinetic effects also play an important role in Cl incorporation. However, further studies are required to clarify the underlying mechanism.

The possible relationship between the surface morphology and residual impurity concentrations was also examined by comparing epilayers grown under different conditions. Although the surface morphology varied with the growth conditions, no systematic relationship was found between the surface morphology and the residual [Si] or [Cl]. This suggests that the residual impurity concentrations are not primarily correlated with the morphology of the flat regions.

To verify that the substantial reduction in both the residual [Si] and [Cl] achieved by eliminating additional HCl also resulted in a low carrier concentration, *C*–*V* and *J*–*V* measurements were performed on a SBD. A schematic cross-sectional structure of the SBD is shown in Figure 7(a), and an optical microscope image of the Schottky anode is shown in Figure 7(b). The homoepitaxial layer was grown under the same conditions as those of the sample shown in Figure 6(b), yielding [Si] <1 × 10^15^ cm^−3^ and [Cl] = 2 × 10^15^ cm^−3^.

**Figure 7.**
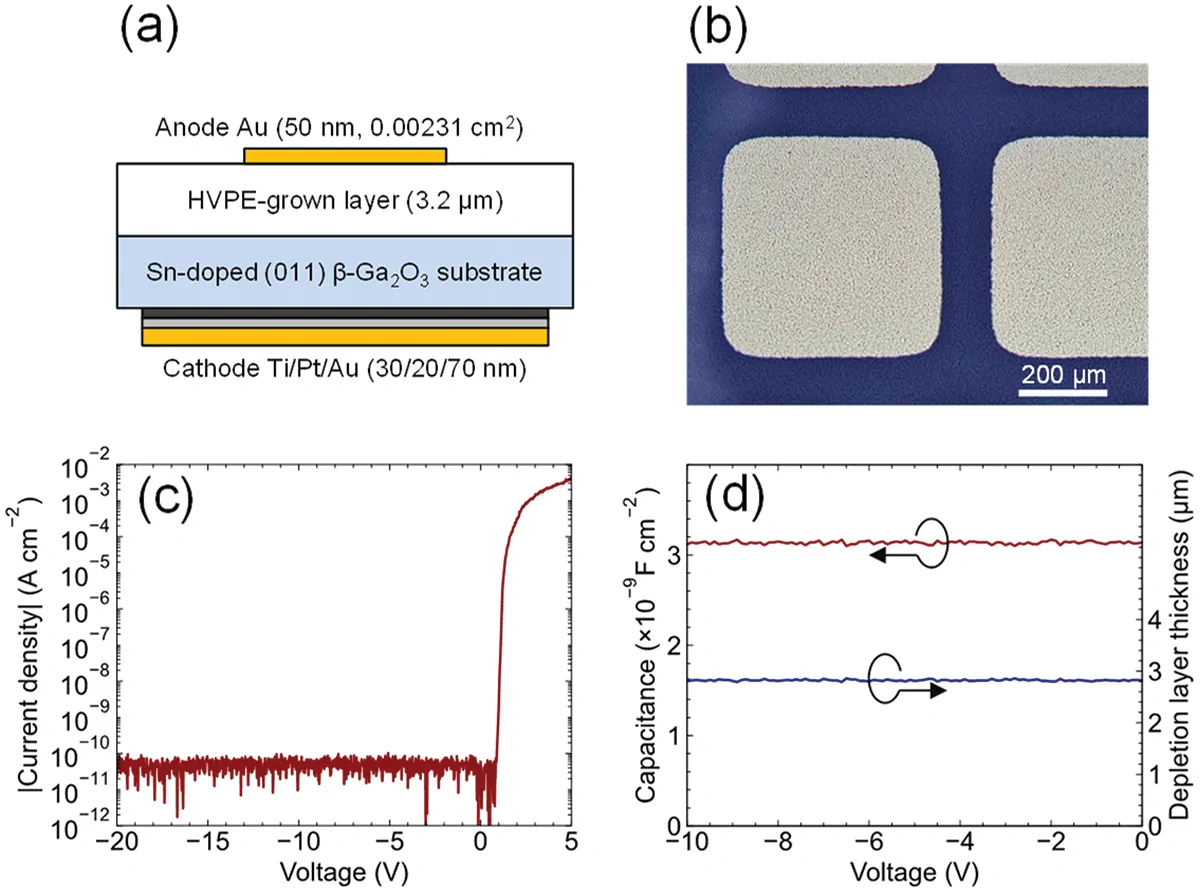
(a) Cross-sectional structure of the SBD used for the *J–V* and *C–V* measurements. (b) Optical microscope image of the Schottky anode. (c), (d) measured *J–V* and *C–V* characteristics, respectively.

Figure 7 shows the *J*–*V* characteristics of the SBD. The reverse leakage current was low, indicating good barrier characteristics. From the slope of the forward *J*–*V* curve above the turn-on voltage, the series resistance was estimated to be approximately 360 kΩ, indicating that the homoepitaxial layer was highly resistive. Because of this high series resistance, a substantial fraction of the applied voltage is expected to drop across the epilayer. Therefore, the forward *J*–*V* characteristics are not suitable for conventional extraction of the ideality factor and Schottky barrier height.

Figure 7 shows the *C*–*V* characteristics of the SBD at 1 MHz. The capacitance showed little dependence on the applied voltage. The depletion width estimated directly from the measured capacitance was approximately 3 μm, which is nearly equal to the epilayer thickness, indicating that the epilayer was nearly fully depleted. To examine whether the capacitance measurement was affected by the high series resistance or by insufficient response of slowly responding electrically active states at 1 MHz, *C*–*V* measurements were also performed at a lower frequency of 100 kHz. Similar voltage-independent capacitance and a depletion width close to the epilayer thickness were obtained at 100 kHz, consistent with the results obtained at 1 MHz. Assuming a built-in potential of 1 V, the *N*_D_ – *N*_A_ required for complete depletion of the epilayer at this voltage was estimated to be at most 1.2 × 10^14^ cm^−3^.

The estimated *N*_D_ − *N*_A_ was considerably lower than the residual [Cl] of approximately 2 × 10^15^ cm^−3^. The activation state of the residual Cl cannot be determined from the present results, and this discrepancy may arise from incomplete donor activation, compensation by unintentional acceptors, or both.

During the investigation of the dependence of the residual impurity concentrations on *P*_0_(HCl), we found that additional HCl plays a critical role in pit formation on the epilayer surface. Figure 8(a) shows a Nomarski optical micrograph of a homoepitaxial layer grown for 1 h at a growth rate of 4.3 μm/h with *P*_0_(GaCl) = *P*_0_(HCl) = 25 Pa and *P*_0_(O_2_) = 2.5 kPa. Numerous pits are observed, with a density of approximately 3.5 × 10^2^ cm^−2^. In contrast, no pits were observed in the homoepitaxial layer shown in Figure 8(b), which was grown for 1 h under the same precursor partial pressures without additional HCl at a comparable growth rate of 4.6 μm/h. Figure 8(c) shows a sample initially grown under the pit-free conditions used for Figure 8(b). After the epilayer thickness reached 2.3 μm, additional HCl was introduced so that the growth conditions became identical to those used for the sample shown in Figure 8(a), and growth was continued until the total thickness reached 4.5 μm. Nevertheless, almost no pits were observed. It should be noted that the residual [Si] in the upper part of the epilayer in Figure 8(c) should be the same as that in the epilayer shown in Figure 8(a), because they were grown under the same additional-HCl condition. Nevertheless, almost no new pits were formed in the epilayer shown in Figure 8(c). This result suggests that the increased Si incorporation itself is not the direct cause of pit formation. These results strongly suggest that pit formation is not caused by the presence of additional HCl during epitaxial growth itself, but rather by the pre-growth etching of the substrate surface by additional HCl before the introduction of GaCl.

**Figure 8.**
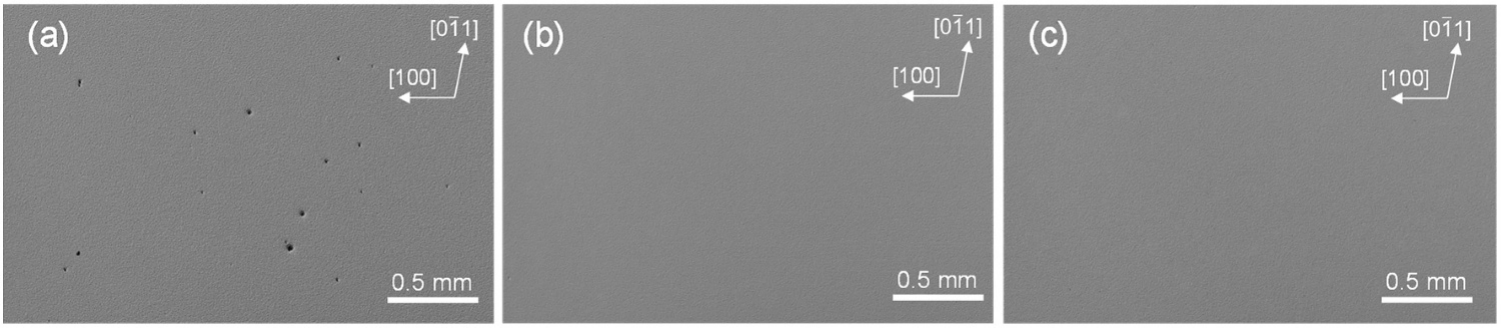
Nomarski optical micrographs of the surfaces of (011) β-Ga_2_O_3_ homoepitaxial layers grown (a) with additional HCl, (b) without additional HCl, and (c) with additional HCl introduced after an initial growth period without additional HCl.

Figure 9(a) shows a Nomarski optical micrograph of a (011) β-Ga_2_O_3_ substrate subjected only to HCl pre-etching under the same conditions as those used prior to the growth of the pit-containing sample shown in Figure 8(a). To more clearly reveal the surface morphology produced by etching, the etching time was increased to 50 min, which is ten times longer than that used in the growth experiments. Numerous hillocks with a density of approximately 1.1 × 10^4^ cm^−2^ were observed, as indicated by the arrows.

**Figure 9.**
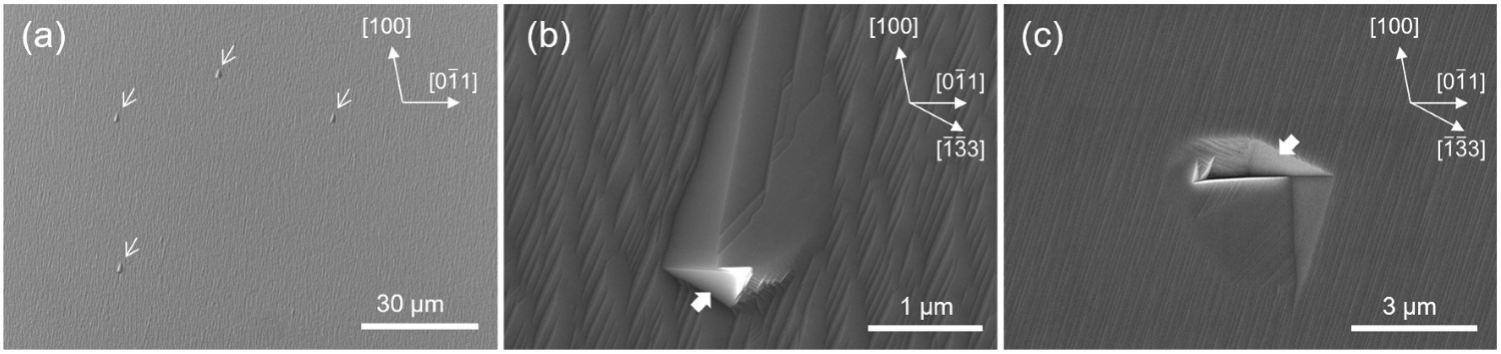
(a) Nomarski optical micrograph and (b) plan-view SEM image of an HCl-etched (011) β-Ga_2_O_3_ substrate. (c) Plan-view SEM image of a pit formed on a (011) β-Ga_2_O_3_ homoepitaxial layer during growth with additional HCl.

Figure 9(b) shows a plan-view SEM image of one of these hillocks. An inclined facet plane, indicated by the arrow, is clearly visible. The trace direction of the facet on the (011) surface coincides with the intersection direction between the (011) and ($\bar{3}$10) planes, i.e. the [$\bar{1}$$\bar{3}$3] direction. Furthermore, the inclination angle of the facet, determined geometrically from the apparent change in facet width in tilted-view SEM observations, was 50.9°, which agrees almost exactly with the theoretical angle of 51.0° between the (011) and ($\bar{3}$10) planes. These observations indicate that the facet is the ($\bar{3}$10) plane. The ($\bar{3}$10) plane is known to be one of the relatively stable low-surface-energy planes of β-Ga_2_O_3_ [37], and the formation of similar ($\bar{3}$10) facets has also been reported during selective HCl etching of (010) and (001) β-Ga_2_O_3_ substrates [38,39].

Figure 9 shows a plan-view SEM image of a pit formed on the homoepitaxial layer grown under additional-HCl supply. Nearly all of the observed pits were accompanied by the ($\bar{3}$10) facet, as indicated by the arrow. These results strongly suggest that HCl pre-etching of the (011) substrate produces stable ($\bar{3}$10) facets, which subsequently act as the origin of pit formation. Owing to their high stability, the growth rate on the ($\bar{3}$10) facets is presumably lower than that on the surrounding (011) surface. Consequently, the growth front above the facets lags behind the surrounding epilayer during homoepitaxy, while the ($\bar{3}$10) facets continue to develop, eventually leading to pit formation.

The dislocation density in EFG-grown β-Ga_2_O_3_ has been reported to be on the order of 10^3^ cm^−2^ [20], which is reasonably close to the hillock density observed after HCl pre-etching. This may indicate that dislocations are involved in hillock formation. On the other hand, the pit density on the homoepitaxial surface was only approximately 3.5 × 10^2^ cm^−2^, much lower than the hillock density. This suggests that only a fraction of the hillocks develop into pits, possibly because only those with particularly well-developed ($\bar{3}$10) facets continue to evolve during subsequent epitaxial growth.

## Conclusions

In summary, we experimentally clarified the effects of *P*_0_(O_2_) and *P*_0_(HCl) on the residual [Si] and [Cl] in (011) β-Ga_2_O_3_ homoepitaxial layers grown by HCl-HVPE. the residual [Si] exhibited a characteristic dependence on *P*_0_(O_2_), decreasing sharply with a relatively small increase in *P*_0_(O_2_) and subsequently remaining nearly constant. In addition, the residual [Si] was reduced below the SIMS detection limit (1 × 10^15^ cm^−3^) in the absence of additional HCl, despite the presence of H_2_ generated during GaCl formation, whereas it increased markedly with increasing *P*_0_(HCl). In contrast, the residual [Cl] showed little dependence on either *P*_0_(O_2_) or *P*_0_(HCl) and remained below 2 × 10^15^ cm^−3^ throughout this study.

To explain these experimental observations, the thermodynamic driving force for SiO_2_ etching was analyzed using the equilibrium parameter *P*_tot_. The calculations revealed that the etching driving force of HCl is much less temperature dependent than that of H_2_ under O_2_-free conditions, allowing HCl to maintain a high etching capability even in the relatively low-temperature source zone (610°C). In contrast, the addition of a small amount of O_2_ dramatically suppressed the etching driving force for both etchants, predicting that SiO_2_ etching in the growth zone is much weaker than that by additional HCl in the source zone. These predictions consistently explain the experimentally observed strong correlation between the residual [Si] and *P*_0_(HCl), the absence of detectable Si without additional HCl despite the presence of H_2_, and the characteristic dependence of the residual [Si] on *P*_0_(O_2_).

Furthermore, by eliminating additional HCl and increasing *P*_0_(GaCl), a growth rate of 13 μm/h, comparable to that previously reported for HVPE with additional HCl, was achieved without particle formation or increases in the residual [Si] and [Cl]. *C*–*V* measurements of a SBD fabricated on such a homoepitaxial layer confirmed its semi-insulating nature, confirming that the residual carrier concentration was successfully reduced, although carrier compensation by unintentionally incorporated acceptors, such as nitrogen, is suggested. We also found that pit formation was dramatically suppressed in the absence of additional HCl and showed that the pits most likely originate from substrate pre-etching by additional HCl, which produces stable ($\bar{3}$10) facets before epitaxial growth.

Although HCl-HVPE of (011) β-Ga_2_O_3_ has been recognized as a promising technique capable of much higher growth rates than Cl_2_-HVPE, its practical application has been hindered by high residual [Si] and pit formation. The present study demonstrates that both of these issues can be effectively overcome. These findings establish HCl-HVPE as a practical route for the high-rate growth of high-quality (011) β-Ga_2_O_3_ homoepitaxial layers and are expected to contribute significantly to the cost-effective manufacturing and widespread deployment of high-performance β-Ga_2_O_3_ power devices.

## Supplementary Material

Supplemental Material
