# Supplementary material for: Rapid growth of a pit-free (011) β-Ga2O3 homoepitaxial layer with very low residual Si and Cl concentrations by HCl-based halide vapor phase epitaxy
Source: Sci Technol Adv Mater. 2026 Oct 22;27(1):2751773. doi: 10.1080/14686996.2026.2751773 (PMC13757811; doi:10.1080/14686996.2026.2751773)
Supplement: Supplementary file 1 [file TSTA_A_2751773_SM8234.docx]

Figure S1. Calculated temperature dependences of the equilibrium partial pressures of Si-containing gaseous species and the etching driving force indicator, *P*_tot_, for SiO_2_ etching by H_2_ under various *P*_0_(O_2_)

Figure S2. Calculated temperature dependences of the equilibrium partial pressures of Si-containing gaseous species and the etching driving force indicator, *P*_tot_, for SiO_2_ etching by HCl under various *P*_0_(O_2_)
